# Primitive neuroectodermal tumor in the spinal canal: A case report

**DOI:** 10.3892/ol.2015.2907

**Published:** 2015-01-27

**Authors:** XIAO-TONG MENG, SHI-SHENG HE

**Affiliations:** Department of Orthopaedics, Shanghai Tenth People’s Hospital Affiliated to Tongji University, Shanghai 200072, P.R. China

**Keywords:** primitive neuroectodermal tumor, chemotherapy, spinal tumor

## Abstract

Primitive neuroectodermal tumors (PNETs) are rare tumors of uncertain histogenesis that occur predominantly in children and young adults. The current study reports a case of PNET in a 60-year-old female, which presented clinically as an intraspinal tumor, causing symptoms of lower back pain, numbness and pain in the right lower extremity. The patient underwent tumorectomy. Following primary therapy, the symptoms of spinal cord compression were relieved. The patient underwent several courses of radiotherapy following surgery but refused to continue with chemotherapy. After a further four months, the tumors recurred and the patient succumbed to the disease.

## Introduction

Primitive neuroectodermal tumors (PNETs) are extremely rare tumors which typically arise from multipotent progenitor cells and are considered to be of neuroectodermal derivation (1). Since the first description of the condition by Hart and Earle in 1973 (2), <100 cases have been documented. The annual incidence of this condition is estimated to range from 0.2–0.4 cases per 100,000 (3), and its concept has been controversial for over a decade, as diagnosis remains difficult and no effective treatment has been identified. PNETs are rapidly growing soft tissue masses, which cause symptoms of nerve compression and pain. They are highly malignant and invasive, with a high rate of relapse and poor prognosis. The five-year survival rate is 30–40% and has not altered significantly over the last 30 years (4,5). PNET primarily occurs in children and young adults, usually <35 years of age, with a mean age of 20 years; however, it may occur at any age, in any population and on any limb (6).

The present study reports the case of an otherwise healthy 60-year-old female with extradural PNET extending into the lumbar cavity, which caused symptoms of nerve root compression. Few such presentations have been reported previously (6–8). Written informed consent was obtained from the patient’s family.

## Case report

**Figure 1:**
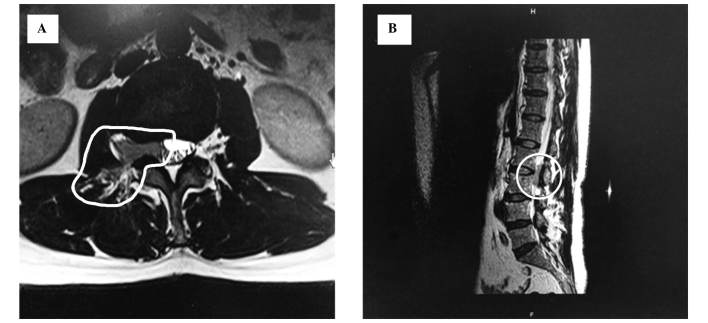
Contrast enhanced magnetic resonance imaging of the lumbar spine revealed (A) a 4.9×2.1×1.8 cm homogeneously enhanced and (B) ill-defined mass from vertebrae L2–L3.

A 60-year-old female was admitted to Shanghai Tenth People’s Hospital Affiliated to Tongji University (Shanghai, China) due to a one-year history of increasing lower back pain, worsening numbness and pain on the back of the right thigh. The patient did not smoke or consume alcohol, and was suffering from diabetes, which was managed by a controlled diet. Initially, the patient’s blood pressure was 130/80 mmHg (normal range, 100–130/60–90 mmHg), with a regular pulse of 80 beats/min (normal range, 60–100 beats/min) and a respiratory rate of 18 breaths/min (normal range, 20–40 breaths/min). The patient was afebrile. On physical examination, hypoesthesia was detected on the back of the thigh and the knee jerk reflex was found to be reduced. In the straight leg raising test an angle of 30° was achieved. The results of all laboratory tests, including complete blood count, renal, bone, hepatic, and coagulation profiles, lactate dehydrogenase, carcinoembryonic antigen, α-fetoprotein, carbohydrate antigen (CA) 19-9 and CA12-5 levels, were normal. Computed tomography (CT) imaging of the lumbar spine revealed a 4.9×2.1×1.8 cm mass with ill-defined margins from the L2–L3 vertebrae (Fig. 1). The lesion was isointense on the T1-weighted image and iso- to hyperintense on the T2-weighted image. Abdominal and pelvic CT imaging and chest X-ray did not reveal any lesions. No evidence of ascites, or retroperitoneal or mesenteric lymphatic metastases was observed.

The patient subsequently underwent intraspinal neoplasm resection. Following a half laminectomy of vertebrae L2 and L3, the 4.9×2.1×1.8 cm ill-defined mass was revealed. The tumor was adhered to the right L2 nerve root and extended into right lumbar cavity through the dilated intervertebral foramen between L2 and L3. Following the complete removal of the mass, the L2 nerve root was dissociated. L3 nerve root activity was improved and all resection margins were free from tumor cells. Macroscopically, the outer surface of the resected mass was white, irregular, firm, and marked with several prominences, and the cut section revealed irregular, ulcerated and necrotic tissue. Postoperatively, the patient showed partial improvement of the deficits; lower back and right thigh pain was alleviated and a significant improvement was achieved in the straight leg raising test, when compared with that observed preoperatively.

Immunohistochemical evaluation of CD99 expression revealed characteristic reactivity on the tumor cell membranes. Histopathological examination of the mass revealed that the tumor cells had small round or oval morphology, strong staining for neuron specific enolase (NSE), fine chromatin, indistinct nucleoli and signs of mitosis. Homer-Wright rosettes were observed, composed of tumor cells surrounding a central region of filamentous fibers combined with necrotic blood vessel and tumor tissue. This led to a diagnosis of PNET.

Focal radiation therapy to the spinal cord (L1–L5) (total dose, 50.4 Gy) was administered over a period of six weeks, however, chemotherapy was refused. Four months following surgery and radiotherapy, the tumors recurred and the patient succumbed to the disease four months later.

## Discussion

PNETs may be classified as central (cPNET) or peripheral (pPNET), and both types have poor prognoses (9). pPNET refers to a group of malignant cells with similar morphological and cytological characteristics and gene expression, that occur in the extracranial soft tissue of the skeletal system (6). This includes several conditions: Ewing’s sarcoma (ES), which occurs intraosteally and extraskeletally; pPNET, which occurs in soft tissue such as paravertebral, abdominal, pelvic or retroperitoneal areas; and Askin tumors, which commonly arise in the chest area (10–12). pPNET and ES are closely related malignancies with small round cells observable on histological examination (13). pPNETs and ES strongly express the glycoprotein p30/32 (CD99), which is encoded by the microneme protein 2 (MIC2) gene. CD99 is expressed in almost all cases in a characteristic membranous manner, however, it is not specific to this condition. The majority of PNET tumor cells stain with vimentins, and neural markers, such as NSE, are frequently expressed (14). PNET has also been shown to stain with keratin in certain cases. Due to their immunohistochemical, ultrastructural and molecular similarities, pPNETs and ES have been categorized into the Ewing family of tumors, which share the same chromosomal translocation but differ in their degree of neural differentiation (15).

Primary spinal PNETs are now more frequently diagnosed due to the improvement of histological techniques, particularly immunohistochemistry. Initially, these cases were diagnosed as astrocytoma, neurofibroma or ependymoma dependent upon on the location of the tumor in relation to the dura (6). Neurofibroma was highly suspected in the current case due to the expansion of the mass into the lumbar cavity through intervertebral foramina; neurofibromas have favorable prognosis if fully excised.

pPNET is a highly aggressive cancer associated with poor prognosis. Surgical excision cannot remove the lesion completely, leading to a postoperative recurrence rate of 90%. The current recommended treatment for pPNET is a combined therapy including surgery, high-dose local radiotherapy and chemotherapy (16).

Although primary PNETs may be indicated by radiological features, immunohistochemical evaluation following surgery is required to confirm the diagnosis. Histological examination must be conducted in every suspected case to diagnose this type of tumor. The current study illustrates a unique case of intraspinal PNET that does not match the typical age distribution for this condition; few similar cases have been previously reported (6–8). Radiological findings in combination with histological examination must be used to differentiate between PNETs and short duration paraparesis.

Despite improvements in surgical techniques and adjuvant chemotherapy, the prognosis of this malignancy remains poor. Further research into the molecular pathogenesis of PNET and its potential application in immunotherapy is crucial for the development of more effective treatments for this condition.

## References

[1] Rorke LB (1983). The cerebellar medulloblastoma and its relationship to primitive neuroectodermal tumors. J Neuropathol Exp Neurol.

[2] Hart MN, Earle KM (1973). Primitive neuroectodermal tumors of the brain in children. Cancer.

[3] Valle JW, Eatock M, Clueit B (2014). A systematic review of non-surgical treatments for pancreatic neuroendocrine tumors. Cancer Treat Rev.

[4] Pape UF, Böhmig M, Berndt U (2004). Survival and clinical outcome of patients with neuroendocrine tumors of the gastroenteropancreatic tract in a german referral center. Ann N Y Acad Sci.

[5] Marinsek ZP, Kavalar R, Jereb B (2006). Ewing sarcoma/PNET: 27 years of experience in Slovenia. Pediatr Hematol Oncol.

[6] Patnaik A, Mishra SS, Mishra S, Deo RC (2013). Review of spinal neuroectodermal tumor. Br J Neurosurg.

[7] Virani MJ, Jain S (2002). Primary intraspinal primitive neuroectodermal tumor (PNET): a rare occurrence. Neurol India.

[8] Nayak PK, Rao KM, Sahoo GC, Mahapatra AK (2011). Primary thoracic primitive neuroectodermal tumor mimicking as neurofibroma. J Neurol India.

[9] Kampman WA, Kros JM, De Jong TH, Lequin MH (2006). Primitive neuroectodermal tumours (PNETs) located in the spinal canal; the relevance of classification as central or peripheral PNET: case report of a primary spinal PNET occurrence with a critical literature review. J Neurooncol.

[10] Khong PL, Chan GC, Shek TW (2002). Imaging of peripheral PNET: common and uncommon locations. Clin Radiol.

[11] Dehner LP (1986). Peripheral and central primitive neuroectodermal tumors. A nosologic concept seeking a consensus. Arch Pathol Lab Med.

[12] Winer-Muram HT, Kauffman WM, Gronemeyer SA, Jennings SG (1993). Primitive neuroectodermal tumors of the chest wall (Askin tumors): CT and MR findings. AJR Am J Roentgenol.

[13] Hisaoka M, Hashimoto H, Murao T (1997). Peripheral primitive neuroectodermal tumour with ganglioneuroma-like areas arising in the cauda equina. Virchows Arch.

[14] Nutman A, Postovsky S, Zaidman I (2007). Primary intraspinal primitive neuroectodermal tumor treated with autologous stem cell transplantation: case report and review of the literature. Pediatr Hematol Oncol.

[15] Kumar R, Reddy SJ, Wani AA, Pal L (2007). Primary spinal primitive neuroectodermal tumor: case series and review of the literature. Pediatr Neurosurg.

[16] Shah JP, Jelsema J, Bryant CS (2009). Carboplatin and paclitaxel adjuvant chemotherapy in primitive neuroectodermal tumor of the uterine corpus. Am J Obstet Gynecol.

